# Information needs of people who have suffered a stroke or TIA and their preferred approaches of receiving health information: A scoping review

**DOI:** 10.1177/23969873241272744

**Published:** 2024-08-26

**Authors:** Jasmin Helbach, Falk Hoffmann, Nina Hecht, Christoph Heesen, Götz Thomalla, Denise Wilfling, Anne Christin Rahn

**Affiliations:** 1 Department of Health Services Research, School of Medicine and Health Sciences, Carl von Ossietzky Universität Oldenburg, Oldenburg, Germany; 2 Nursing Research Unit, Institute of Social Medicine and Epidemiology, University of Lübeck, Germany; 3 Institute of Neuroimmunology and Multiple Sclerosis (INIMS), Center for Molecular Neurobiology, University Medical Center Hamburg-Eppendorf, Hamburg, Germany; 4 Department of Neurology, University Medical Center Hamburg-Eppendorf, Hamburg, Germany

**Keywords:** Information needs, stroke, information seeking behavior

## Abstract

**Purpose:**

We aimed to synthesize the information needs of people with stroke (PwS) in recurrent stroke prevention.

**Methods:**

In this scoping review we searched Medline (via PubMed), CINAHL, and PsycINFO from inception to June 5, 2023, to identify all studies describing the information needs of people 18 years and older who have suffered a stroke or transient ischemic attack within the past 5 years. We included qualitative and quantitative studies from developed countries published in German or English. Data analysis was performed following Arksey and O’Malley’s methodological framework for scoping reviews.

**Findings:**

We screened 5822 records for eligibility and included 36 articles published between 1993 and 2023. None of the included studies used a comprehensive framework or defined information needs. Based on statements from PwS and their caregivers, PwS needed information on treatment, etiology, effects of stroke, prognosis, rehabilitation, discharge, life changes, care role, support options, information sources, and hospital procedures. The most frequently expressed needs were information on the treatment (77.8%) and stroke etiology (63.9%). The primary information source was healthcare professionals (85.7%), followed by written information (71.4%), family and friends (42.6%), and the internet (35.7%), with information provided directly by healthcare professionals being preferred. The timing of information transfer is often described as too early.

**Conclusion:**

PwS are primarily interested in clinical information about stroke, for example, treatment and etiology, and less often in information about daily life, for example, rehabilitation, the role of care, or lifestyle changes. PwS prefer to receive information directly from healthcare professionals. Developing a shared understanding of PwS’s information needs is crucial to implement suitable strategies and programs for dealing with these needs in clinical practice.

## Graphical abstract

**Graphical abstract:**
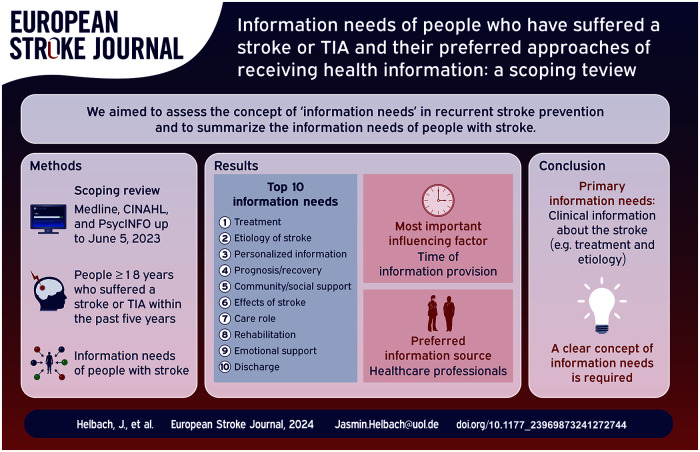

## Background

Stroke is one of the leading causes of mortality and morbidity in industrialized countries with significant public health importance.^1^ In 2019, there were more than 12 million incident cases of stroke and more than 6 million deaths worldwide. This makes stroke the second leading cause of death and the second leading cause of disability, with 143 million disability-adjusted life years lost.^1^

People who have suffered a stroke or transient ischemic attack (TIA), summarized as people with stroke (PwS), are at high risk of having another stroke. The recurrence rates increased from 1.2% within the first 30 days to 7.4% within the first year and 19.4% in the following 5 years.^2^ After the acute stroke treatment of stroke,^3,4^ long-term management for preventing stroke recurrence (e.g. lifestyle changes and pharmacotherapy) represents a crucial aspect of stroke management.^5,6^ However, PwS show low adherence to some of the preventive measures, particularly the taking of medication.^7^ Several factors contribute to treatment adherence, such as social support, cognitive and emotional dysfunction, physician-patient communication, and knowledge about medication effects, benefits, and harms.^8–10^ To improve adherence, it is essential to empower PwS to make informed decisions, ensuring they understand the consequences of the condition and the benefits and risks of treatment options.^11,12^

Long-term management after stroke often presents challenges for PwS due to physical and psychological disabilities and concerns.^13–15^ To support self-management, healthcare professionals need to support PwS’s gaining knowledge about life after stroke through targeted, patient-centered information provision.^16^ In this context, the empowerment process, that is, actively enabling PwS to make independent health-related decisions, must be oriented toward the needs of PwS. However, the concept of “information needs” is very complex and influenced by individual goals, contextual and social conditions.^17,18^ To meet their needs, PwS used and prioritize different sources of information.^19,20^

Existing evidence syntheses mainly focus on the general needs of PwS^21–23^ and contain little specific insights on information needs. However, evidence suggests that information needs are among the most important unmet needs.^21,23^ Apart from one review on the information needs and information behavior of patients and relatives in general acute care, including stroke as a medical condition,^24^ there is no evidence synthesis on the information needs of PwS.

Therefore, this scoping review aims to synthesize the information needs described by PwS. In addition, we will investigate whether the needs change over the course of the disease, which information sources are used and preferred, and which contextual factors influence the information needs of PwS.

## Methods

We used a scoping review approach to overview the concepts and major domains of “information needs” in recurrent stroke prevention. We followed the methodological framework for scoping reviews described by the Joanna Briggs Institute (JBI)^25^ and reported according to the extension of the Preferred Reporting Items for Systematic Reviews and Meta-Analysis statement for scoping reviews (PRISMA-ScR).^26^ The protocol was registered in the Open Science Framework (https://osf.io/2tja8/).

### Inclusion criteria

We included qualitative studies with a minimum sample size of ⩾8 participants and quantitative studies with a sample size of ⩾30 participants. Studies not published in English or German were excluded due to a lack of language skills. No restrictions were placed on the study’s duration or date. We used the PCC (population, concept, context) approach for scoping reviews recommended by the JBI^25^ to determine further eligibility criteria (see below).

#### Population

We included studies of participants 18 years and older who have suffered a stroke or TIA within the past 5 years to ensure that they could remember and recall their needs during and after a stroke. As PwS are often dependent on support and unable to express their needs independently, we also included studies that focused on informal caregivers of PwS. Studies focusing on the perspective or information needs of health professionals were excluded.

#### Concept

We included studies focusing on the information needs of PwS and their informal caregivers. Information needs were defined as personally expressed needs that are not defined by an expert but result from the patients’ “*recognition that their knowledge is inadequate to satisfy a goal, within the context/situation that they find themselves at a specific point in the time*” *[pg 92]*^17^ according to the definitions of Timmins^27^ and Ormandy.^17^ Here, the experts’ central task is to support patients in formulating and specifying information needs.^17^ When searching for information, individuals may interact with various manual or web-based information systems (e.g. face-to-face information, prints like flyers or brochures, or the World Wide Web). In studies, the information needs are typically assessed through qualitative interviews or questionnaires asking participants if they are satisfied with the information provided or need information on a particular topic. We excluded studies only reporting on satisfaction of information received, general needs (such as health service and care needs), or lifestyle change interventions without mentioning information needs related to PwS.

#### Context

As information needs may change depending on the situation, contextual conditions, and course of disease, we included studies regardless of the setting (e.g. clinical, rehabilitation, or community). We considered both the acute phase of the event, when PwS are still in hospital, as well as the post-discharge phase (up to 6 weeks), the rehabilitation phase (up to 6 months) and the post-stroke phase (more than 6 months-up to 5 years). If it was not possible to assign the described needs to a specific phase, or if several phases were addressed, the study was classified as “Period after stroke not specified.” As this scoping review is carried out as part of a research project on the medical prevention of recurrent stroke in Germany,^28^ we excluded studies from developing countries, defined according to the Official Development Assistance (ODA) lists of the Development Assistance Committee (DAC).^29^

### Search strategy and study selection

We searched the electronic bibliographic databases Medline (via PubMed), CINAHL, and PsycINFO from inception to June 5, 2023. The search strategy, based on two search strings for “stroke” and “information needs” is presented in the Supplemental Material (sTable1). Furthermore, we manually screened the reference lists of included studies and performed a forward-citation search for potentially relevant articles using Web of Science on September 26, 2023.

We transferred the identified studies to the Endnote 20 library (version 20, Clarivate, Philadelphia, PA, USA) and, after removing all duplicates, to Covidence (Covidence systematic review software, Veritas Health Innovation, Melbourne, Australia), an online review management tool. Two reviewers (JH, NH, DW, or AR) independently screened all titles and abstracts against the specified inclusion criteria. To facilitate adequate agreement, we pilot-tested on 50 articles. Afterward, the full texts of all eligible records were screened independently by two reviewers from the same research team based on the same inclusion criteria. We piloted the full-text screening on five full-texts. Any disagreements were resolved by discussion or by a third reviewer (JH, DW, or AR).

### Data extraction

Characteristics of the included studies (author, year of publication, country, study design, study duration, and aim of the study), the participants (number, sex, age, diagnosis, degree of disability, living environment and the period after stroke), as well as the methodological approaches of data collection (data collection procedures, instruments used), were extracted by one reviewer (JH) and verified by a second reviewer (AL). In addition, specific information on the concept of information needs (definition, components of information needs), the areas of information needs, the type of information seeking (active searching, passive searching, ongoing searching, and passive attention), the intervening variables (psychological, role-related or interpersonal, environmental or source characteristics), and the used and preferred sources of information were extracted by one reviewer (JH) and verified by a second reviewer (NH). All data were first extracted in a standardized piloted extraction form (available on request from the authors) before being transferred to the figures and tables presented in the article.

### Quality assessment

In accordance with the guidance for conducting scoping reviews,^25^ and the general aim of scoping reviews to provide a comprehensive overview of the available evidence, regardless of its quality, no quality assessment was performed.

### Deviation from the protocol

All content and procedures described in the protocol were generally followed, but minor adjustments were made. First, to clarify the focus of the study, we removed the differences between populations and settings from our objectives and included the intervening factors instead. Nevertheless, all information (including populations and settings) were extracted as planned and reported in the Results Section. Second, the population was described in the protocol as PwS and their family members. In the manuscript, we have used the term “informal caregivers” rather than “family members” in line with the included studies. This term is slightly broader and would also include close relatives and very close friends.

### Data synthesis

We analyzed the data according to the methodological framework for scoping reviews by Arksey and O’Malley.^30^ As a first step, we descriptively analyzed baseline characteristics, including size, nature, and the included studies’ empirical and analytical methods. Secondly, we described the “information needs” concepts used in the studies. In the final step, we extracted and visualized the information needs in a mind map. Overlaps between the studies were discovered and key domains were identified. Then, we assessed the importance of each domain of information needs based on their frequencies, examined whether and how the needs changed during the course of the disease, and which information-seeking approaches were preferred. The results were synthesized by one reviewer (JH) and discussed with another reviewer (AR).

## Results

### Study selection

**Figure 1.**
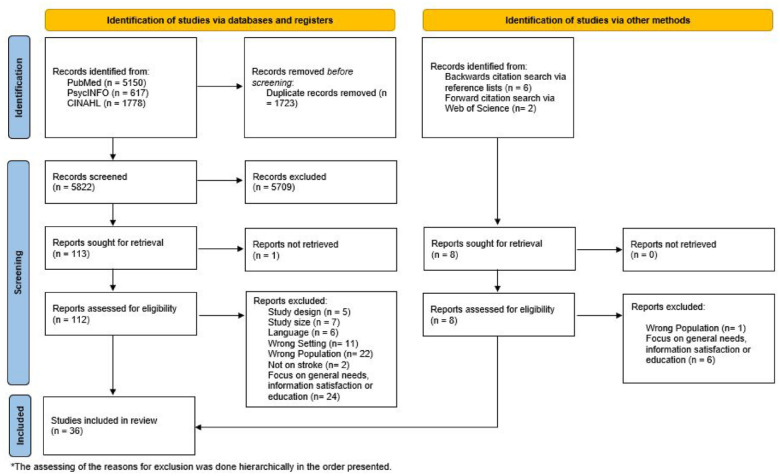
Flow chart.

The systematic literature search identified a total of 5822 records (Figure 1). After screening title and abstract, we read the full texts of 113 articles classified as potentially relevant, of which one could not be retrieved.^31^ We included 35^32–66^ articles. By searching forward and backward, we identified eight additional articles, of which one^67^ was included (36 articles in total). All articles were written in English and published between 1993 and 2023. Supplemental Material (sTable2) shows all excluded records after the full-text screening, with reasons for exclusion.

### Study and participant characteristics

The characteristics of all 36 included studies are shown in Table 1. Ten studies were conducted in the UK (27.7%), followed by seven studies from Australia (19.4%), six from the US (16.7%), five from Sweden (13.8%), three from Canada (8.3%), two from The Netherlands (5.5%), and one each (2.7%) from Finland, Ireland, Scotland, and New Zealand. Twenty-five (69.4%) studies used a qualitative design, nine (25.0%) were quantitative studies, and two (5.6%) used a mixed-methods approach. Qualitative studies used semi-structured individual interviews (21/25; 84.0%), group interviews (3/25; 12.0%), or both (1/25; 4.0%) to collect data. All quantitative studies were cross-sectional and used surveys (5/9; 55.6%) or standardized interviews (4/9; 44.4%) for data collection. Both mixed-methods studies used semi-structured interviews and combined them once with survey data only (1/2; 50.0%) and once with survey data and art therapy techniques (1/2; 50.0%). The number of participants included ranged from 8 to 243 for qualitative studies, 32 to 630 for cross-sectional studies, and 12 to 14 for mixed-methods studies.

**Table 1.** Characteristics of the included studies.

Author	Country	Study design	Aim	Period after stroke	Setting	Data source	Time of data collection	Number of participants	Age range of included participants; proportion of female
Abrahamson & Wilson^32^	UK	Qualitative research	Explored needs identified by patients, how they were addressed by the six-month review (6MR), and whether or not policy aspirations for the review were substantiated by the data.	No specific time period	Community-based	Semi-structured interviews	2015–2016	Stroke patients: 46	Patients: 28–91 years; n.a.
Allison et al.^33^	UK	Qualitative research	Explore the experiences of patients and carers receiving secondary prevention advice and use these to inform the development of an educational resource.	No specific time period	Community-based	Semi-structured interviews; Focus groups	n.a.	Stroke patients: 25; Caregivers: 13	Patient: 37–91 years; female 44.0%^a^
									Caregivers: n.a.; female 61.5%^a^
Almborg et al.^34^	Schweden	Cross-sectional studies	Describe stroke patients’ perceptions of their participation in discharge planning and identify the correlates of perceived participation.	After discharge (up to 6 weeks)	Community-based	Standardized interviews	October 2003–November 2005	Stroke patients: 188	Patients: ⩽65 = 20.7%, 65–74 = 26.1%, 75–84 = 34.5%, ⩾85 = 18.6%; female 44.1%^b^
Almborg et al.^35^	Schweden	Cross-sectional studies	Describe relatives’ perceptions of participation in discharge planning and identify correlates associated with the relatives’ perceived participation.	After discharge (up to 6 weeks)	Community-based	Standardized interviews	October 2003–November 2005	Stroke-Caregivers dyads: 152	Patients: ⩽65 = 22%, 65–74 = 28%, 75–84 = 32%, ⩾85 = 28%; female 43%
									Caregivers: ⩽54 = 34%, 54–65 = 26%, 65–74 = 27%, ⩾75 = 12%; female 68%
Bakas et al.^36^	US	Qualitative research	Determine the self-reported needs, concerns, strategies, and advice of family caregivers of stroke survivors during the first 6 months after hospital discharge.	Rehabilitation (first 6 months)	Community-based	Semi-structured Interviews	n.a.	Caregivers: 14	Caregiver: n.a.; female 100%
Bamm et al.^37^	Canada	Qualitative research	Explore current models of delivery of rehabilitation services from the perspectives of patients, families, and healthcare providers	Rehabilitation (first 6 months)	Rehabilitation	Semi-structured interviews	10-month period in 2011	Stroke patients: 8; Caregivers: 4	Patients: 19–86 years; female 62.5%^c^
									Caregivers: Female 75%^c^
Berg et al.^38^	Finland	Cross-sectional studies	Study the information needs of the spouses of stroke survivors, and whether the functional ability, depressive mood, or demographic factors of the survivors or spouses associate with the information needs or satisfaction with care and whether prescheduled follow-up improves information provision.	No specific time period	n.a.	Standardized interviews	1 January –30 June 2010, 2 January–30 June 2012	Caregivers: 96	Caregivers: 2010: mean 64.2 years (SD 12.7), female 64%, 2012: mean 66.9 (SD 9.5), female 54%
								2010, n = 59	
								2012, n = 37	
Camicia et al.^39^	US	Mixed methods	Explore the needs of family members of stroke patients admitted to an inpatient rehabilitation facility (IRF)	No specific time period	Rehabilitation	Semi-structured interviews; Art therapy technique; survey	n.a.	Caregivers: 12	Caregivers: 18–45: 25%, 46–65: 50%, 66–85: 25%; female 75%
Cobley et al.^40^	UK	Qualitative research	Investigate patients’ and carers’ experiences of Early Supported Discharge services and inform future Early Supported Discharge service development and provision	Post-stroke (more than 6 months)	Community-based	Semi-structured interviews	n.a.	Stroke patients: 27; Caregivers 15	Patients: mean 69.9 years (SD 13.42); female n.a.
									Caregivers: mean 72.79 years (SD: 14.10); female 87%
Croot et al.^41^	UK	Qualitative research	Explore patients’ experience and response to TIA and to any care they received as a result of the TIA	No specific time period	Community-based	Semi-structured interviews	March–July 2011, April–July 2012	Stroke patients: 39	Patients: 31–89 years; female 46.2%^d^
Dalli et al.^42^	Australia	Cross-sectional studies	Investigate whether survivors of stroke/TIA understand explanations about their prescribed prevention medications and their associations with medication adherence, control of risk factors, and unmet needs.	Post-stroke (more than 6 months)	Community-based	Survey	December 2018	Stroke patients: 630	Patients: Median 69.4 (IQR 61.2–76.7); female 36.8%
Decker et al.^43^	US	Qualitative research	Define the information needs and preferred presentation format for stroke survivors, caregivers, and clinicians considering r-tPA treatment	Acute phase (staying at the stroke unit)	Hospital	Focus group	n.a.	Stroke patients: 39; Caregivers 24	Patients: Mean 58.9 years; female 57%
Donnellan et al.^44^	Ireland	Qualitative research	Interpret the explanations that patients gave of their experience after stroke and how these may validate an already established patient-focused intervention framework – the Quest for quality and improved performance (QQUIP) (2006)	Rehabilitation phase (first 6 months)	n.a.	Semi-structured interviews	n.a.	Stroke patients: 8	Patients: 52–83 years; female 25%^e^
Eames et al.^45^	Australia	Qualitative research	To identify the preferences of patients with stroke and their carers for format and delivery style, of different categories of stroke information and whether these preferences changed over time.	Rehabilitation phase (first 6 months)	n.a.	Semi-structured interviews	November 2007–June 2008	Stroke patients: 34; Caregivers 18	Patients: mean 63 years, range 28–85 years; female 41.2%
									Caregivers: mean 58 years, range 26–77; female 72.2%
Finch et al.^46^	Australia	Qualitative research	Explore the perceptions of stroke survivors and carers toward stroke education in an Australian health context.	No specific time period	Community-based	Focus group	November 2018–June 2019	Stroke patients: 15; Caregivers 4	Patients: n.a.; female 46.7%^f^
									Caregivers: n.a.; female 50%^f^
Garrett and Cowdell^47^	UK	Qualitative research	Discover the perceived information needs of patients and carers at 2, 20, and 90 days post-stroke.	No specific time period	More than one setting	Semi-structured Interviews	n.a.	Stroke patients: 16	n.a.
Hanger et al.^48^	New Zealand	Qualitative research	Clarify what issues are important to stroke patients and their carers. To determine whether these issues change over time.	No specific time period	Community-based	Semi-structured interviews	n.a.	Stroke patients: 243	Patients: 23–101 years; female 44.9%^g^
Hinojosa and Rittman^49^	US	Cross-sectional studies	Examine the association between health education needs and physical injury sustained as a result of activities related to the caregiving role	Post-stroke (more than 6 months)	Community-based	Telephone survey	May 2006-September 2007.	Caregivers: 276	Caregivers: Mean 65.5 years, range 18–88 years; female 90.9%
Hinojosa and Rittman^50^	US	Cross-sectional studies	Highlight the need for information dissemination to Puerto Rican and Mainland caregivers within the VHA system	Post-stroke (more than 6 months)	Community-based	Telephone survey	n.a.	Caregivers: 120	Caregivers: Mean 61.6 years (SD 11.6); female 92.5%
Hoffmann et al.^51^	Australia	Qualitative research	(a) Determine current practice in the provision of written information to stroke patients and their carers (b) Explore their informational needs while in hospital and 6 months later (c) Examine the suitability of the written materials received, comparing readability levels to participants’ general reading ability.	No specific time period	More than one setting	Semi-structured interviews	n.a.	Stroke patients: 57; Caregivers 12	Patients: mean 72.2 years (SD 13.4), range 35–92; female 47.4%
									Caregivers: mean 61.3 years (SD 11.4), range 42–83; female 75%
Kerr et al.^52^	UK	Qualitative research	(a) Determine what information people who have had a stroke would like to see on a website about living with stroke (b) Determine the most effective means of structuring information on the website c) Identify any differences between people with and without aphasia in terms of preferences for structuring information on the website	Post-stroke (more than 6 months)	Community-based	Focus groups	n.a.	Stroke patients: 12	Patients: Mean 67.8 years (SD 12.8), range 45–86; female 58.3%
Kristensson and Björkdahl^53^	Sweden	Qualitative research	The objective of the study was to explore how relatives of stroke survivors perceived the information provided by the stroke unit	No specific time period	More than one setting	Semi-structured interviews	March 2015–September 2015	Caregivers: 14	Caregivers: mean 65 years, range 31–78; female 85.7%^h^
Lamontagne et al.^54^	Canada	Qualitative research	This study reports perceptions of persons with stroke and their caregivers in an existing continuum of stroke care, social services, and rehabilitation in the Province of Quebec	Post-stroke (more than 6 months)	Community-based	Focus groups	n.a.	Stroke patients: 37; Caregivers 31	Patients: mean 59.6 years (SD 11.6), range 39–85; female 43.2%^i^
									Caregivers: mean 58.8 years (SD 15.1), range 17–82; female 74.2%^i^
O’Connell et al.^55^	Australia	Qualitative research	Determine caregivers’ perspective of their support and educational needs within the acute hospital and community setting.	No specific time period	More than one setting	Semi-structured interviews	n.a.	Caregivers: 28	Caregivers: mean 54.93 years (SD 12.38), range 23–74; female 57.1%^j^
Perry and Middleton^56^	Australia	Cross-sectional studies	To advance understanding of stroke care-giving in Australia, using assessment methods and tools previously used successfully elsewhere	Rehabilitation phase (first 6 months)	Community-based	Standardized interviews	May–July 2006	Patient -caregiver dyads: 32	Patients: Sydney (ASU): mean 66.4 (SD 16.0); female 70.0%^k^
								Sydney (ASU): *n* = 10	Sydney (post): mean 62.8 (SD 12.7); female 27.3%^k^
								Sydney (post) *n* = 11	Brisbane (ASU): mean 60.1 (SD 19.2); female 45.5%^k^
								Brisbane (ASU) *n* = 11	Caregivers: Sydney (ASU): mean 57 (SD 7.3); 50.0%^k^
									Sydney (post): mean 63.8 (SD 15.9); 72.7%^k^
									Brisbane (ASU): mean 56.7 (SD 15.0); female 45.5%^k^
Rosenthal et al.^67^	US	Mixed-methods	Identify the perceived needs of wives of hospitalized stroke patients and then determine the level of importance of those needs and the degree to which the wives felt the hospital nursing staff met their needs.	After discharged (up to 6 weeks)	Rehabilitation	Self-administered questionnaire; Semi-structured interviews	n.a.	Caregivers: 14	Caregivers: Mean 60.1 years (SD 10.5), range 39–77; female 100%
Souter et al.^57^	UK	Qualitative research	Explore stroke patients’ and carers’ beliefs and concerns about medicines and identify the barriers to medication adherence for secondary stroke prevention	No specific time period	Community-based	Semi-structured interviews	n.a.	Stroke patients: 30	Patients: 32–86 years; female 50.0%^l^
Tooth and Hoffmann^58^	Australia	Qualitative research	Explore the extent, source and format of the information received by stroke rehabilitation patients and their perceptions of the quality of that information. Additionally, the readability levels of the written materials. received were assessed	Rehabilitation phase (first 6 months)	Community-based	Semi-structured interviews	n.a.	Stroke patients: 15	Patients: Mean 68 years (SD 18); female 47%
van der Smagt-Duijnstee et al.^59^	Netherlands	Qualitative research	Explore the experiences and needs of relatives during the entire hospitalization periods of hospitalized stroke patients.	Acute phase (staying at the stroke unit)	Hospital	Semi-structured interviews	November 1996–April 1997	Caregivers: 17	Caregivers: mean 63 years, 39–84; female 70.6%^m^
van der Smagt-Duijnstee et al.^60^	Netherlands	Cross-sectional studies	Explore the experiences and needs of relatives of hospitalized stroke patients.	Acute phase (staying at the stroke unit)	Hospital	Survey	n.a.	Caregivers: 106	Caregivers: mean 56 years (SD 14); female 66%
van Veenendaal et al.^61^	Canada	Cross-sectional studies	Identify informational needs of stroke survivors and their family members as perceived by themselves and by health professionals. The source of information and the desired source for future information were also explored.	No specific time period	n.a.	Survey	n.a.	Stroke patients: 35; Caregivers: 39	Patients: mean 61 years, range 36–79; n.a.
									Caregivers: mean 62 years, range 36–84; n.a.
Visvanathan et al.^62^	UK	Qualitative research	Explore decision-making regarding stroke treatments that increased the likelihood that the patient who has been left with significant disability as a result of stroke will survive longer	No specific time period	Hospital	Semi-structured interviews	September 2017–January 2018; follow up April 2018–July 2018	Stroke patients: 15	Patients: Mean 79 years, range 53.93; female 60%^n^
von Vogelsang et al.^63^	Sweden	Qualitative research	To describe patients’ perceived and expected recovery 1 year after aneurysmal subarachnoid hemorrhage (aSAH)	Post-stroke (more than 6 months)	Community-based	Semi-structured interviews	March–December 2015	Stroke patients: 16	Patients: Median 51 years, IQR 41–65; female 75%
Wallengren et al.^64^	Sweden	Qualitative research	To explore relatives’ information needs and the characteristics of their information-seeking process shortly after the stroke event and 6 months later.	No specific time period	more than one setting	Semi-structured interviews	August 2003–September 2004	Caregivers: 16	Caregivers: Mean 58 years, range 30–79; female 81.3%
Wellwood et al.^65^	Scotland	Qualitative research	Discover the service users’ (patients’ and carers’) perceptions and knowledge of their illness	After discharged (up to 6 weeks)	Community-based	Semi-structured interviews	n.a.	Stroke patients: 65; Caregivers: 80	Patients: mean 69 years (SD 14.1); female 48%
									Caregivers: n.a.
Wiles et al.^66^	UK	Qualitative research	Identify a range of information needs that patients and carers may have at three different phases post-stroke: during hospitalization; up to 1 month post-discharge, and 2 months to 1 year poststroke	No specific time period	more than one setting	Semi-structured interviews	n.a.	Stroke patients 9; Caregivers: 2	Patients: 50–85 years^+^; female 47.6%^o^
								Patient-caregiver dyads: 10	Caregivers: n.a.

R-QPD: Relative’s questionnaire about participation in discharge planning; P-QPD: patients’ questionnaire on participation in discharge planning.

a Own calculation based on the numbers provided by the authors in Table 1: Patients (11/25); Caregivers (8/13).

b Own calculation based on the numbers provided by the authors in Table 2 (83/188).

c Own calculation based on the numbers provided by the authors in the text: patients (5/8); caregivers (3/4).

d Own calculation based on the numbers provided by the authors in the text (18/39).

e Own calculation based on the numbers provided by the authors in Table 2 (2/8).

f Own calculation based on the numbers provided by the authors in the text: patients (7/15); caregivers (2/4).

g Own calculation based on the numbers provided by the authors in Table 1 (109/243)

h Own calculation based on the numbers provided by the authors in the text (12/14).

i Own calculation based on the numbers provided by the authors in Table 2 patients (16/37); caregivers (23/31).

j Own calculation based on the numbers provided by the authors in the text (16/28).

k Own calculation based on the numbers provided by the authors in Table 1: patient Sydney (ASU) (7/10), Sydney (post) (3/11), Brisbane (ASU) (5/11); caregivers Sydney (ASU) (5/10), Sydney (post) (8/11), Brisbane (ASU) (5/11).

l Own calculation based on the numbers provided by the authors in Table 1 (15/30).

m Own calculation based on the numbers provided by the authors in the text (12/17).

n Own calculation based on the numbers provided by the authors in Table 2 (9/15).

o Own calculation based on the numbers provided by the authors in the text (10/21).

+ Including patients who were not directly involved in the study, but for whom the caregivers were interviewed (total *n* = 21).

One-third each of the 36 included studies involved only PwS, only caregivers, or both PwS and caregivers. Three studies (8.3%) focused on the acute phase immediately after the stroke when the PwS are still in hospital, four studies (11.0%) on the first six weeks, six (16.7%) on the first 6 months, and seven studies (19.4%) on the post-stroke phase when the stroke occurred at least 6 months ago. The period after stroke was not specified in 16 studies (44.4%). Most studies (19/32, 52.8%) were conducted in a community setting. The mean age for PwS ranged from 59 to 79 (*n* = 11 studies), and for caregivers, from 58 to 72 years (*n* = 15 studies). The proportion of females ranged from 25% to 75% (*n* = 21 studies) among PwS and 50% to 100% among caregivers (*n* = 19 studies).

### Concept of “information needs”

None of the included studies used a comprehensive framework nor defined information needs.

### Information needs

**Figure 2.**
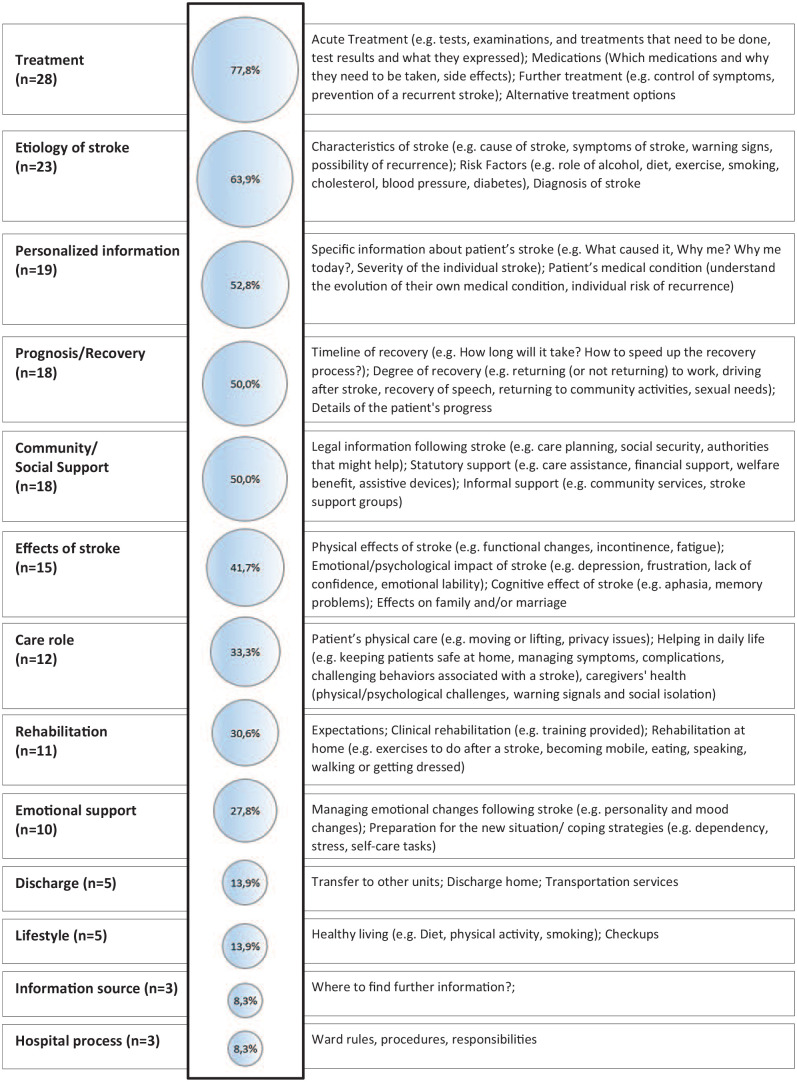
Percentage of studies describing the individual domains of information needs and the content covered.

Based on the studies included, we identified 14 main domains of information needs, illustrated in Figure 2 and specified in the Supplemental Material (sTable 3).

The most frequently mentioned need for information was related to treatment. 77.8% (28/36) of the included studies mentioned this domain and reported a need for information on acute treatment (11/28; 39.3%), drug treatment (10/28; 35.7%), and long-term treatment to reduce symptoms and prevent further strokes (13/28; 46.4%). The second most frequently mentioned need for information concerned the etiology of the stroke, which was stated in 63.9% of the studies (23/36). Most studies described the need for more information on stroke characteristics (17/23; 73.9%) and risk factors (5/23; 21.7%). Few described a need for information on the diagnosis (3/23; 13.0%), general symptoms (2/23; 8.7%), or warning signs (1/23; 4.35%). Additionally, there was a great need for personalized information about the patient’s stroke (19/36; 52.8%), prognosis (18/36; 50.0%), particularly about the degree of recovery (7/18; 38.9%) and the timeline of recovery (6/18; 33.3%), as well as information on possible effects of stroke on life (15/36; 41.7%). Here, the need for information about the physical (6/15; 40.0%), emotional (6/15; 40.0%), cognitive (4/15; 26.7%), and behavioral effects (2/15; 13.3%) was described. Additionally, four studies (4/15; 26.7%) described the need for information about the impact of stroke on family and marriage.

The need for information on community and social support was described in 50.0% (18/36) of the studies, with a wide range of different needs expressed regarding legal information after a stroke (8/18; 44.5%), statutory support (10/18; 55.6%), and informal support options (3/18; 16.7%). In addition, a need for information about emotional support options was described in 27.8% (10/36) of the studies, including specific information on coping strategies to deal with the emotional ups and downs after a stroke (7/10; 70.0%), as well as on processing the event and raising awareness of the situation they now find themselves in (2/10; 20.0%).

In 33.3% (12/36) of the studies, information was requested that relates to the role of caregivers, including information on how to care for PwS at home (5/12; 41.7%) and how to support them with physical care (4/12; 33.3%). The need for information about rehabilitation was mentioned in 30.6% (11/36) of the studies, with a need for information about rehabilitation at home (5/11; 45.5%), expectations of rehabilitation (3/11; 27.3%), and rehabilitation facilities (1/11; 9.1%). Information needs on transport and discharge, recommended lifestyle changes, possible sources of information, and general information about the hospital process were described in less than 20% of studies.

### Comparison of the information needs of PwS and caregivers

Distinguishing between studies including only PwS (*n* = 12) and those including only caregivers (*n* = 12) reveals some differences in the prioritization of information needed. In studies including only PwS, the need for information about treatments (8/12; 66.7%) and the etiology of stroke (7/12; 58.3%) was most frequently described. In studies including only caregivers, the greatest need for information was described in relation to the role of caregiving (9/12; 75.0%), which was not mentioned by any of the studies including only PwS. Caregivers’ need for information about treatment was 66.7% (8/12), followed by the need for information about community and social support (7/12; 58.3%), which was mentioned in 25.0% (3/12) of the studies focusing on PwS. None of the studies including only caregivers described a need for information about transfer and discharge, compared with four studies including only PwS (4/12; 33.3%).

### Information needs during the course of a stroke

The time after stroke was defined in 20 of the included studies, with three referring to the acute phase, four to the discharge phase, six to the rehabilitation phase, and seven to the post-stroke phase (sTable 4). All studies related to the acute phase reported a need for information on the treatment and effects of stroke and most additionally reported a need for personalized information and information on the etiology of stroke. In the discharge phase, additional information on the role of care and rehabilitation was needed. The need for information about community and social support increased over the course of disease. Some information needs such as transfer and discharge, lifestyle and other information sources were only mentioned from the rehabilitation phase onward.

### Used and preferred sources of information

A total of 14 studies described the sources of information used, and ten studies included an indication of the preferred sources of information for PwS and caregivers. The most commonly used source of information was healthcare professionals (12/14; 85.7%), followed by written information such as brochures, leaflets or books (10/14; 71.4%). The Internet (5/14; 35.7%) and the social network of friends and family (6/14; 42.6%) were also used as sources of information. A mixed format of oral and written information given by and discussed with healthcare professionals was preferred.

### Intervening variables

**Figure 3.**
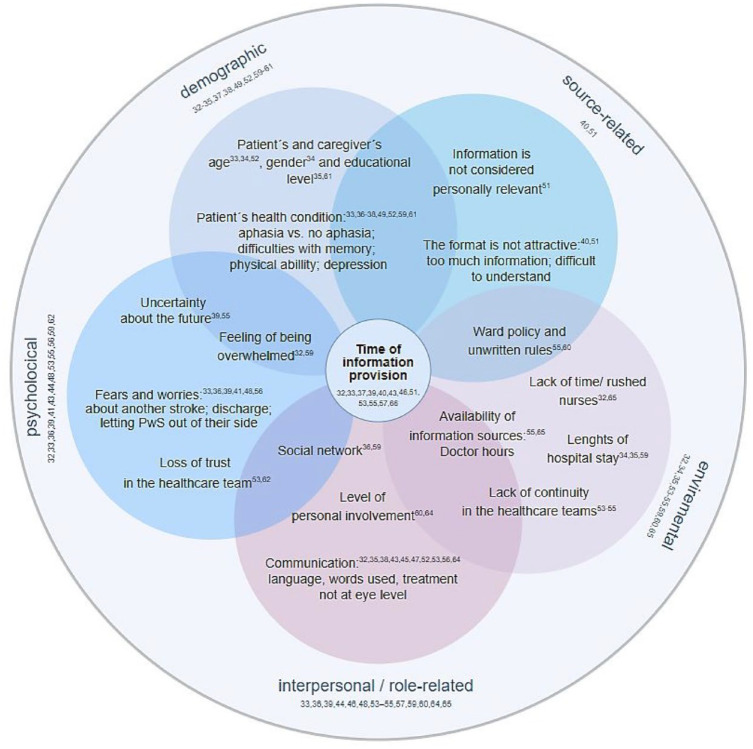
Factors influencing the information needs of PwS and caregivers.

Overall, a wide range of intervening factors that increase the information needs of PwS were described in the included studies, some of which were interrelated (Figure 3). The most important factor related to all dimensions of the intervening variables was the timing of information provision (12/36; 33.3%), which was generally considered too early. Of the 36 included studies, 38.9% (14/36) described factors related to the interpersonal context. The most common factor considered here, and most often rated as inadequate, was the communication between the healthcare professionals and the PwS (10/14; 71.4%), including the choice of words, technical language and treatment not at eye level. Factors attributable to the psychological context were described in 36.1% (13/36) of the included studies. Here, fears and worries about suffering another stroke (4/13; 30.8%), uncertainty about the future (2/13; 15.4%), feeling overwhelmed (3/13; 23.1%), and loss of trust in the treatment team (2/13; 15.4%) were described. The influence of demographic factors such as age, gender, level of education, or state of health was reported in 11 of the 36 studies (30.6%). The environmental context was described as an influencing factor in 25.0% (9/36). A lack of continuity in the nursing team (3/9; 33.3%), a lack of time and thus rushed nursing staff and restless wards (2/9; 22.2%) and the availability of doctors as a source of information (2/9; 22.2%) were considered here. Two of the 36 studies (5.6%) described factors relating to the source of information, including the length (2/2; 100%) and complexity (1/2; 50%) of the information material.

## Discussion

In this scoping review, which includes 36 studies, we found a wide range of information needs among PwS and their caregivers. PwS and caregivers are primarily interested in treatment, stroke etiology, and personalized information about the cause of the patient’s stroke. Information that is important for daily living, such as rehabilitation, prognosis, role in care, and social participation, received less attention. Additionally, we found a large number of different variables influencing PwS’s information needs. The most frequently described influencing factor was the timing of the information provision, which was usually considered too early. None of the studies provided a concept of what is understood by “information needs.” Our findings demonstrate the complexity of the phenomenon and suggest that a wide range of personal and environmental factors influences information needs.

According to the information needs outlined in the studies included in this review, PwS and their caregivers are most interested in general information about the treatment, etiology, and cause of their stroke, rather than information relevant to life after a stroke. Similar trends have been observed in reviews of other clinical conditions, such as cancer,^68–70^ depression and anxiety,^71^ or other cardiovascular diseases.^72,73^ These reviews also suggest that information needs change over time. In addition, only a few studies focused on a specific time period after the stroke, making it difficult to determine whether certain needs become more important at different times during the course of the stroke. However, the included studies that refer to a specific time window indicate that some information needs, such as information on prognosis, transportation and discharge or emotional support, become more important during the rehabilitation and post-stroke phase (at least 6 weeks after the stroke). It is striking that although almost no study focuses explicitly on the acute phase, the greatest need for information relates to the clinical information on the treatment and etiology of stroke. Several studies have reported that clinical information was given during the acute event when PwS could not process it and could not remember the exact details.^32,33,37,39,43,51,55,57^ It is likely that other topics, such as rehabilitation, are discussed later in the course of the disease and are, therefore, better remembered. Also, considering demographic factors is highly relevant as those influence individual needs.^33–35,52,61^ For example, age is an important factor in how people want to participate in medical decisions. One included study found that older age is associated with less involvement in decision-making and concluded that healthcare professionals should pay more attention to patients over the age of 85 during discharge planning.^34^ Another study indicated, that PwS reported feeling too old to change their lifestyle.^33^ Thus, it might be supportive to implement structures to identify and address individual patient needs at different time points during their hospital stay and to mention topics to be discussed at later time points in referral letters.

We found that PwS use various sources to obtain the information they need but prefer to receive information directly from their physicians and nurses. This is in line with other research^69,74–77^ showing that healthcare professionals have been and still are the most trusted source of information. However, healthcare professionals often have limited time-resources to meet this demand, which may increase the need for information. Particularly concerning clinical information, PwS rely on healthcare professionals to share information. PwS often do not feel adequately informed due to a lack of access to and communication with healthcare professionals.^33,36,44,46,48,54,56,57,65^ They reported disappointment,^53,62^ a feeling of being overwhelmed^32,53^ unprepared^39^ or abandoned,^44^ which may have led to unmet clinical information needs being better remembered and mentioned more frequently in a survey or interview. However, the included studies also indicate that PwS prefer a combination of written and oral information and are open to using different resources, including the Internet.^46,47,50,54,58^ To reduce dissatisfaction and better respond to patients’ needs, it may be possible in the future to utilize eHealth solutions additionally. A recent review in the field of oncology suggests that the proactive promotion of digital information sources and their integration into the clinical process by healthcare professionals could offer potential benefits to patients.^78^ EHealth solutions, such as apps, can provide access to medical knowledge, facilitate communication between patients and health professionals, increase adherence to therapy, and empower patients to manage their care.^79–83^ A scoping review on mobile health interventions delivers first indications that medication adherence and physical activity might be improved as essential aspects of recurrent stroke prevention such as changes in risk factors, lifestyle behaivior and adherence to medication.^84^ However, the endpoint recurrent stroke was only assessed in one study, and no difference was found. Another potential form of support is peer support. Peer support offers emotional and informational assistance, helping individuals understand the illness and strengthening their self-esteem.^85–87^ Self-help groups have been mentioned as a useful or preferred source of information by some of the included studies.^36,37,46,58^ In other studies, a need for information on support groups was described.^51,52,58^ Health professionals play an essential role in addressing the identified needs. However, many of these needs do not necessarily require the expertise of the physician. Physicians should, therefore focus primarily on discussing the patient’s clinical data and utilize the trust placed in them to actively direct PwS to other sources of information, for example, offers from health insurance companies (including apps) or local self-help support groups.

Regardless of the information source, the material must be designed to be understandable for PwS. Provided information is often difficult to understand and is not perceived as personally relevant or attractive to read.^40,51^ In particular, understanding information about the long-term consequences, such as medication and treatment options to reduce the risk of recurrent stroke, appears to be challenging. As information is often only provided on request,^36,37,40,53,55,65^ which may lead to PwS being insufficiently informed, active carefully-dosed information provision emerges as a potential solution.^88^ The provision of patient decision aids that provide basic information about a condition or treatment in plain language can assist PwS in improving their general knowledge about the disease, risk factors, and the influence of lifestyle factors. By sharing evidence-based patient information,^89,90^ PwS can be empowered to understand their health condition, develop questions and needs, address them in communication with healthcare professionals, and make informed decisions. Some approaches already rely on evidence-based patient information to increase PwS involvement in decision-making.^91,92^ While using an SDM encounter tool improved patient involvement,^92^ no effects on adherence were shown after 10 months.^93^ Here, additional interventions such as motivational interviewing after a decision-making process might be helpful to support adherence by addressing individual needs in managing medications. Although the evidence in stroke research is limited,^94^ several reviews on other chronic illnesses,^95–97^ particularly cardiovascular diseases,^95^ have demonstrated large clinical benefits of motivational interviewing.

### Limitations

To the best of our knowledge, this is the first scoping review examining the information needs of PwS. Nevertheless, some limitations occurred. As a further limitation the search strategy was limited to articles published in English and German, which may lead to the exclusion of potentially relevant articles. In addition, articles from developing countries defined according to the Official Development Assistance (ODA) lists of the Development Assistance Committee (DAC)^29^ were excluded, which limits the generalizability of the results. It should be noted that articles from countries with high publication rates, such as China or Korea, were listed and excluded accordingly. Due to the lack of a general concept of information needs, it was difficult to clearly identify whether studies reflect information needs or only the experiences and satisfaction of PwS. However, to identify as many relevant studies as possible, we independently screened all studies dealing with information needs, information-seeking behavior or information sources and conducted a forward and backward search. We included both qualitative and quantitative studies in our review, which made data synthesis more difficult but also expanded the range of data. We did not perform a quality assessment of the included studies. However, this is in line with the guidelines for conducting scoping reviews^25^ and is based on the general aim of the scoping review to consider all available evidence.

## Conclusion

This scoping review provides a comprehensive overview of PwS’s information needs based on statements from themselves and their caregivers. The most frequently needed information is clinical information about the stroke, for example, treatment and etiology, while information about daily life, for example, rehabilitation, the role of care, or lifestyle changes, is less frequently needed. PwS often experience anxiety and worry due to early, overwhelming, and poorly communicated information, preferring stepwise direct communication with healthcare providers in combination with written materials. Support interventions should be based on an individual needs assessment, and timing should be considered an essential factor in delivering person-centered interventions responding to individual information needs.

## Supplemental Material

sj-docx-1-eso-10.1177_23969873241272744 – Supplemental material for Information needs of people who have suffered a stroke or TIA and their preferred approaches of receiving health information: A scoping review Supplemental material, sj-docx-1-eso-10.1177_23969873241272744 for Information needs of people who have suffered a stroke or TIA and their preferred approaches of receiving health information: A scoping review by Jasmin Helbach, Falk Hoffmann, Nina Hecht, Christoph Heesen, Götz Thomalla, Denise Wilfling and Anne Christin Rahn in European Stroke Journal
