# Supplementary material for: Information needs of people who have suffered a stroke or TIA and their preferred approaches of receiving health information: A scoping review
Source: Eur Stroke J. 2024 Aug 26;10(1):5–21. doi: 10.1177/23969873241272744 (PMC11569532; doi:10.1177/23969873241272744)
Supplement: sj-docx-1-eso-10.1177_23969873241272744 – Supplemental material for Information needs of people who have suffered a stroke or TIA and their preferred approaches of receiving health information: A scoping review [file sj-docx-1-eso-10.1177_23969873241272744.docx]

**Supplement**

1. sTable1 Search strategies
2. sTable 2 Excluded studies with reasons for exclusion
3. sTable 3 Detailed overview of the frequency of the domains of information requirements and their subdomains with corresponding references
4. sTabele 4 Information needs stratified by time period after the stroke (n=20)
5. References

**sTable1 Search strategies**

| **Database** | **Search strategy** |
| --- | --- |
| **PubMed** | ("stroke"[MeSH Terms] OR "ischemic attack, transient"[MeSH Terms] OR "Intracranial Hemorrhages"[MeSH Terms] OR "stroke"[Title/Abstract] OR "strokes"[Title/Abstract] OR "cerebrovascular accident*"[Title/Abstract] OR "cerebral vascular accident"[Title/Abstract] OR "cva"[Title/Abstract] OR "apoplex*"[Title/Abstract] OR "transient ischemic attack*"[Title/Abstract] OR "transient ischaemic attack*"[Title/Abstract] OR "tia"[Title/Abstract] OR (("brain*"[Title/Abstract] OR "cerebr*"[Title/Abstract] OR "cerebell*"[Title/Abstract] OR "intracr*"[Title/Abstract] OR "intracerebr*"[Title/Abstract]) AND ("ischemi*"[Title/Abstract] OR "ischaemi*"[Title/Abstract] OR "infarct*"[Title/Abstract] OR "thromb*"[Title/Abstract] OR "embol*"[Title/Abstract] OR "occlus*"[Title/Abstract] OR "hypox*"[Title/Abstract])) OR (("brain*"[Title/Abstract] OR "cerebr*"[Title/Abstract] OR "cerebell*"[Title/Abstract] OR "intracr*"[Title/Abstract] OR "intracerebr*"[Title/Abstract] OR "intraventricular"[Title/Abstract] OR "subarachnoid*"[Title/Abstract]) AND ("haemorrhage*"[Title/Abstract] OR "hemorrhage*"[Title/Abstract] OR "haematoma*"[Title/Abstract] OR "hematoma*"[Title/Abstract] OR "bleed*"[Title/Abstract])) OR "SAH"[Title/Abstract] OR "SAHs"[Title/Abstract]) AND ("Personal Satisfaction"[MeSH Terms] OR "Patient Education as Topic"[MeSH Terms] OR "health knowledge, attitudes, practice"[MeSH Terms] OR "Consumer Health Information"[MeSH Terms] OR "Needs Assessment"[MeSH Terms] OR "information need"[Title/Abstract:~5] OR "information needs"[Title/Abstract:~5] OR "information needed"[Title/Abstract:~5] OR "information needing"[Title/Abstract:~5] OR "health literacy"[Title/Abstract] OR "health information"[Title/Abstract:~3] OR "health-related information"[Title/Abstract] OR "disease knowledge"[Title/Abstract:~3] OR "disease understanding"[Title/Abstract:~3] OR "disease understand" [Title/Abstract:~3] OR "disease understood"[Title/Abstract:~1] OR "diagnosis understand"[Title/Abstract:~3] OR "diagnosis understanding"[Title/Abstract:~3] OR "diagnosis understood"[Title/Abstract:~1]) |
| **CINAHL** | (MH ("Stroke+" OR "Cerebral Ischemia, Transient" OR "Intracranial Hemorrhage+") OR (TI ("stroke" OR "strokes" OR "cerebrovascular accident*" OR "cerebral vascular accident" OR "cva" OR "apoplex*" OR "transient ischemic attack*" OR "transient ischaemic attack*" OR "tia") OR AB ("stroke" OR "strokes" OR "cerebrovascular accident*" OR "cerebral vascular accident" OR "cva" OR "apoplex*" OR "transient ischemic attack*" OR "transient ischaemic attack*" OR "tia")) Or ((TI ("brain*" OR "cerebr*" OR "cerebell*" OR "intracr*" OR "intracerebr*" OR "intraventricular" OR "subarachnoid*") OR AB ("brain*" OR "cerebr*" OR "cerebell*" OR "intracr*" OR "intracerebr*" OR "intraventricular" OR "subarachnoid*")) AND (TI ("ischemi*" OR "ischaemi*" OR "infarct*" OR "thromb*" OR "embol*" OR "occlus*" OR "hypox*" OR "haemorrhage*" OR "hemorrhage*" OR "haematoma*" OR "hematoma*" OR "bleed*") OR AB ("ischemi*" OR "ischaemi*" OR "infarct*" OR "thromb*" OR "embol*" OR "occlus*" OR "hypox*" OR "haemorrhage*" OR "hemorrhage*" OR "haematoma*" OR "hematoma*" OR "bleed*"))) OR ( TI ("SAH" OR "SAHs") OR AB ("SAH" OR "SAHs")) ) AND ( MH ("Personal Satisfaction" OR "Consumer Health Information" OR "Needs Assessment" OR "Information Needs") OR (TI ("information need*" OR TI information N5 need OR information N5 needs OR information N5 needed) OR AB ("information need*" OR information N5 need OR information N5 needs OR information N5 needed)) OR (TI "health literacy" OR AB "health literacy") OR (TI (health N3 information OR "health-related information") OR AB (health N3 information OR "health-related information")) OR (TI (disease N3 knowledge OR disease N3 understanding OR disease N3 understand OR disease N1 understood OR diagnosis N3 understand OR diagnosis N3 understanding OR diagnosis N1 understood) OR AB (disease N3 knowledge OR disease N3 understanding OR disease N3 understand OR disease N1 understood OR diagnosis N3 understand OR diagnosis N3 understanding OR diagnosis N1 understood))) |
| **PsycINFO** | ((DE ("Cerebral Hemorrhage" OR "Subarachnoid Hemorrhage" OR "Cerebral Ischemia" OR "Cerebral Infarction") OR MA ("Stroke" OR "ischemic attack, transient" OR "Intracranial Hemorrhages")) OR (TI ("stroke" OR "strokes" OR "cerebrovascular accident*" OR "cerebral vascular accident" OR "cva" OR "apoplex*" OR "transient ischemic attack*" OR "transient ischaemic attack*" OR "tia") OR AB ("stroke" OR "strokes" OR "cerebrovascular accident*" OR "cerebral vascular accident" OR "cva" OR "apoplex*" OR "transient ischemic attack*" OR "transient ischaemic attack*" OR "tia")) OR ((TI ("brain*" OR "cerebr*" OR "cerebell*" OR "intracr*" OR "intracerebr*" OR "intraventricular" OR "subarachnoid*") OR AB ("brain*" OR "cerebr*" OR "cerebell*" OR "intracr*" OR "intracerebr*" OR "intraventricular" OR "subarachnoid*")) AND (TI ("ischemi*" OR "ischaemi*" OR "infarct*" OR "thromb*" OR "embol*" OR "occlus*" OR "hypox*" OR "haemorrhage*" OR "hemorrhage*" OR "haematoma*" OR "hematoma*" OR "bleed*") OR AB ("ischemi*" OR "ischaemi*" OR "infarct*" OR "thromb*" OR "embol*" OR "occlus*" OR "hypox*" OR "haemorrhage*" OR "hemorrhage*" OR "haematoma*" OR "hematoma*" OR "bleed*"))) OR ( TI ("SAH" OR "SAHs") OR AB ("SAH" OR "SAHs")) ) AND ( (DE ("Client Satisfaction" OR "Needs Assessment" OR "Health Literacy") OR MA ("Personal Satisfaction" OR "Patient Education as Topic" OR "health knowledge, attitudes, practice" OR "Consumer Health Information" OR "Needs Assessment") OR (TI ("information need*" OR information Near/5 need OR information Near/5 needs OR information Near/5 needed) OR AB ("information need*" OR information Near/5 need OR information Near/5 needs OR information Near/5 needed)) OR (TI "health literacy" OR AB "health literacy") OR (TI (health Near/3 information OR "health-related information") OR AB (health Near/3 information OR "health-related information")) OR (TI (disease Near/3 knowledge OR disease Near/3 understanding OR disease Near/3 understand OR disease Near/1 understood OR diagnosis Near/3 understand OR diagnosis Near/3 understanding OR diagnosis Near/1 understood) OR AB (disease Near/3 knowledge OR disease Near/3 understanding OR disease Near/3 understand OR disease Near/1 understood OR diagnosis Near/3 understand OR diagnosis Near/3 understanding OR diagnosis Near/1 understood))) |

**sTable 2 Excluded studies with reasons for exclusion**

| Aaby A, Friis K, Christensen B, Rowlands G, Maindal HT. Health literacy is associated with health behaviour and self-reported health: A large population-based study in individuals with cardiovascular disease. *Eur J Prev Cardiol*. 2017;24(17):1880-1888. doi:10.1177/2047487317729538 | Not on stroke (other diseases) |
| --- | --- |
| Abrahamson V, Wilson P. Positioning the six-month review in the recovery process post-stroke: The ideology of personal responsibility. *Health Soc Care Community*. 2019;27(1):249-259. doi:10.1111/hsc.12677 | Focus on general needs, information satisfaction or education |
| Alphonsa A, Sharma KK, Sharma G, Bhatia R. Knowledge regarding oral anticoagulation therapy among patients with stroke and those at high risk of thromboembolic events. *J stroke Cerebrovasc Dis Off J Natl Stroke Assoc*. 2015;24(3):668-672. doi:10.1016/j.jstrokecerebrovasdis.2014.11.007 | Wrong Setting (developing countries) |
| Anderson CS, Linto J, Stewart-Wynne EG. A population-based assessment of the impact and burden of caregiving for long-term stroke survivors. *Stroke*. 1995;26(5):843-849. doi:10.1161/01.str.26.5.843 | Focus on general needs, information satisfaction or education |
| Andreassen S, Wyller TB. Patients' experiences with self-referral to in-patient rehabilitation: a qualitative interview study. *Disabil Rehabil*. 2005;27(21):1307-1313. doi:10.1080/09638280500163711 | Study size (n<8 quali; n<30 quanti) |
| Andrew NE, Kilkenny MF, Naylor R, Purvis T, Cadilhac DA. The relationship between caregiver impacts and the unmet needs of survivors of stroke. *Patient Prefer Adherence*. 2015;9:1065-1073. Published 2015 Jul 27. doi:10.2147/PPA.S85147 | Focus on general needs, information satisfaction or education |
| Bakas T, Jessup NM, McLennon SM, Habermann B, Weaver MT, Morrison G. Tracking patterns of needs during a telephone follow-up programme for family caregivers of persons with stroke. *Disabil Rehabil*. 2016;38(18):1780-1790. doi:10.3109/09638288.2015.1107767 | Focus on general needs, information satisfaction or education |
| Barber N, Parsons J, Clifford S, Darracott R, Horne R. Patients’ problems with new medication for chronic conditions. *Qual Saf Health Care*. 2004;13(3):172-175. doi:10.1136/qhc.13.3.172 | Study size (n<8 quali; n<30 quanti) |
| Bauler S, Jacquin-Courtois S, Haesebaert J, et al. Barriers and facilitators for medication adherence in stroke patients: a qualitative study conducted in French neurological rehabilitation units. *Eur Neurol*. 2014;72(5-6):262-270. doi:10.1159/000362718 | Wrong Population (age (<18), stroke more than 5 years ago, health care professionals) |
| Baumann M, Le Bihan E, Chau K, Chau N. Associations between quality of life and socioeconomic factors, functional impairments and dissatisfaction with received information and home-care services among survivors living at home two years after stroke onset. *BMC Neurol*. 2014;14:92. doi:10.1186/1471-2377-14-92 | Focus on general needs, information satisfaction or education |
| Berry N, Astley C, Du H, et al. Perceptions from Cardiac and Stroke Patients of the Information Provided by Health Professionals at Discharge from Hospital (2012-2014). *Heart Lung Circ*. 2016;25:S301-S301. doi:10.1016/j.hlc.2016.06.709 | Wrong Study design (Conference abstracts, reviews) |
| Beunder C, Dekker JHM, Brasser JA, Depla MFIA. Thuis na een CVA: ‘Dan begint het pas’ Een kwalitatief onderzoek naar de behoefte aan nazorg van CVA-patiënten na terugkeer naar huis = Home after a stroke; “that’s when it really begins” A qualitative study into the needs for aftercare of stroke patients. *Tijdschr Gerontol Geriatr*. 2015;46(4):196-203. doi:10.1007/s12439-015-0139-z | Language (not in English or German) |
| Cecil R, Parahoo K, Thompson K, McCaughan E, Power M, Campbell Y. 'The hard work starts now': a glimpse into the lives of carers of community-dwelling stroke survivors. *J Clin Nurs*. 2011;20(11-12):1723-1730. doi:10.1111/j.1365-2702.2010.03400.x | Wrong Population (age (<18), stroke more than 5 years ago, health care professionals) |
| Cook AM, Pierce LL, Hicks B, Steiner V. Self-care needs of caregivers dealing with stroke. *J Neurosci Nurs J Am Assoc Neurosci Nurses*. 2006;38(1):31-36. doi:10.1097/01376517-200602000-00007 | Wrong Population (age (<18), stroke more than 5 years ago, health care professionals) |
| Crow J. A 2-week stroke review identifies unmet needs in patients discharged home from a hyperacute stroke unit. *Br J Neurosci Nurs*. 2018;14(1):29-35. doi:10.12968/bjnn.2018.14.1.29 | Study size (n<8 quali; n<30 quanti) |
| Danzl MM, Harrison A, Hunter EG, et al. "A Lot of Things Passed Me by": Rural Stroke Survivors' and Caregivers' Experience of Receiving Education From Health Care Providers. *J Rural Health*. 2016;32(1):13-24. doi:10.1111/jrh.12124 | Focus on general needs, information satisfaction or education |
| Davidson CS, Wallace SE. Information needs for carers following a family member’s right hemisphere stroke. *Aphasiology*. 2022;36(3):291-316. doi:10.1080/02687038.2021.1873906 | Study size (n<8 quali; n<30 quanti) |
| Davoody N, Koch S, Krakau I, Hägglund M. Post-discharge stroke patients’ information needs as input to proposing patient-centred eHealth services. *BMC Med Inform Decis Mak*. 2016;16:66. doi:10.1186/s12911-016-0307-2 | Wrong Population (age (<18), stroke more than 5 years ago, health care professionals) |
| de Oliveira BC, Garanhani ML, Garanhani MR. Caregivers of people with stroke - needs, feelings and guidelines provided. *Acta Paul Enferm*. 2011;24(1):43-49. | Study size (n<8 quali; n<30 quanti) |
| De Simoni A, Shanks A, Balasooriya-Smeekens C, Mant J. Stroke survivors and their families receive information and support on an individual basis from an online forum: descriptive analysis of a population of 2348 patients and qualitative study of a sample of participants. *BMJ Open*. 2016;6(4):e010501-e010501. doi:10.1136/bmjopen-2015-010501 | Focus on general needs, information satisfaction or education |
| Denham AMJ, Wynne O, Baker AL, et al. “This is our life now. Our new normal”: A qualitative study of the unmet needs of carers of stroke survivors. *PLoS One*. 2019;14(5):e0216682-e0216682. doi:10.1371/journal.pone.0216682 | Wrong Population (age (<18), stroke more than 5 years ago, health care professionals) |
| Dennehy K, Synnott P, Murphy R, et al. 123 Empowering Patients after Stroke: Development of a Stroke Information Booklet...67th Annual & Scientific Meeting of the Irish Gerontological Society, Innovation, Advances and Excellence in Ageing, 26–28 September 2019, Cork, Ireland. *Age Ageing*. 2019;48:iii17-iii65. doi:10.1093/ageing/afz103.71 | Wrong Study design (Conference abstracts, reviews) |
| Díez-Ascaso O, Martinez-Sánchez P, Fuentes B, Díez-Tejedor E. Sociocultural study on the self-perception of stroke and an analysis of doctor-patient communication. *Neurologia*. 2011;26(2):81-91. doi:10.1016/j.nrl.2010.09.017 | Language (not in English or German) |
| Dudka S, Winczewski P, Janczewska K, Kubsik A, Woldańska-Okońska M. [The education influence on effects of rehabilitation in patients after stroke]. *Pol Merkur Lekarski*. 2016;41(245):225-230. | Language (not in English or German) |
| Eames S, McKenna K, Worrall L, Read S. The suitability of written education materials for stroke survivors and their carers. *Top Stroke Rehabil*. 2003;10(3):70-83. doi:10.1310/KQ70-P8UD-QKYT-DMG4 | Wrong Population (age (<18), stroke more than 5 years ago, health care professionals) |
| Hanger HC, Mulley GP. Questions people ask about stroke. *Stroke*. 1993;24(4):536-538. doi:10.1161/01.str.24.4.536 | Wrong Population (age (<18), stroke more than 5 years ago, health care professionals) |
| Hare R, Rogers H, Lester H, McManus R, Mant J. What do stroke patients and their carers want from community services? *Fam Pract*. 2006;23(1):131-136. doi:10.1093/fampra/cmi098 | Wrong Population (age (<18), stroke more than 5 years ago, health care professionals) |
| Hayashi Y, Hai HH, Tai NA. Assessment of the needs of caregivers of stroke patients at state-owned acute-care hospitals in southern Vietnam, 2011. *Prev Chronic Dis*. 2013;10:E139-E139. doi:10.5888/pcd10.130023 | Wrong Setting (developing countries) |
| Hillsdon KM, Kersten P, Kirk HJS. A qualitative study exploring patients’ experiences of standard care or cardiac rehabilitation post minor stroke and transient ischaemic attack. *Clin Rehabil*. 2013;27(9):845-853. doi:10.1177/0269215513478956 | Focus on general needs, information satisfaction or education |
| Ing MM, Linton KF, Vento MA, Nakagawa K. Investigation of Stroke Needs (INVISION) Study: Stroke Awareness and Education. *Hawai’i J Med public Heal a J Asia Pacific Med Public Heal*. 2015;74(4):141-145. | Focus on general needs, information satisfaction or education |
| Jamison J, Graffy J, Mullis R, Mant J, Sutton S. Barriers to medication adherence for the secondary prevention of stroke: a qualitative interview study in primary care. *Br J Gen Pract J R Coll Gen Pract*. 2016;66(649):e568-76. doi:10.3399/bjgp16X685609 | Wrong Population (age (<18), stroke more than 5 years ago, health care professionals) |
| Jullamate P, de Azeredo Z, Pául C, Subgranon R. Thai stroke patient caregivers: who they are and what they need. *Cerebrovasc Dis*. 2006;21(1-2):128-133. doi:10.1159/000090211 | Wrong Setting (developing countries) |
| Kamalakannan S, Gudlavalleti Venkata M, Prost A, et al. Rehabilitation Needs of Stroke Survivors After Discharge From Hospital in India. *Arch Phys Med Rehabil*. 2016;97(9):1526-1532.e9. doi:10.1016/j.apmr.2016.02.008 | Wrong Setting (developing countries) |
| Keaton L, Steiner V, Masterson M, et al. An E-rehabilitation team helps caregivers deal with stroke. *Internet J Allied Heal Sci Pract*. 2004;2(4):17p-17p. | Focus on general needs, information satisfaction or education |
| Kerr SM, Smith LN. Stroke: an exploration of the experience of informal caregiving. *Clin Rehabil*. 2001;15(4):428-436. doi:10.1191/026921501678310234 | Focus on general needs, information satisfaction or education |
| Kersten P, Low JTS, Ashburn A, George SL, McLellan DL. The unmet needs of young people who have had a stroke: results of a national UK survey. *Disabil Rehabil*. 2002;24(16):860-866. doi:10.1080/09638280210142167 | Wrong Population (age (<18), stroke more than 5 years ago, health care professionals) |
| Khodambashi S, Haugland D, Ellingsberg A, Kottum H, Sund JK, Nytrø Ø. An Experimental Comparison of a Co-Design Visualizing Personal Drug Information and Patient Information Leaflets: Usability Aspects...The 16 World Congress of Medical and Health Informatics: Precision Healthcare Through Informatics (MedInfo2017) was held . *Stud Heal Technol Informatics*. 2017;245:748-752. doi:10.3233/978-1-61499-830-3-748 | Wrong Population (age (<18), stroke more than 5 years ago, health care professionals) |
| Kim JW, Moon SS. Needs of family caregivers caring for stroke patients: based on the rehabilitation treatment phase and the treatment setting. *Soc Work Health Care*. 2007;45(1):81-97. doi:10.1300/J010v45n01_06 | Wrong Setting (developing countries) |
| King RB, Semik PE. Stroke caregiving: difficult times, resource use, and needs during the first 2 years. *J Gerontol Nurs*. 2006;32(4):37-44. doi:10.3928/00989134-20060401-07 | Focus on general needs, information satisfaction or education |
| Kjörk EK, Sunnerhagen KS, Lundgren-Nilsson Å, Andersson AK, Carlsson G. Development of a Digital Tool for People With a Long-Term Condition Using Stroke as a Case Example: Participatory Design Approach. *JMIR Hum factors*. 2022;9(2):e35478-e35478. doi:10.2196/35478 | Wrong Population (age (<18), stroke more than 5 years ago, health care professionals) |
| Krishnan S, Hay CC, Pappadis MR, Deutsch A, Reistetter TA. Stroke Survivors' Perspectives on Post-Acute Rehabilitation Options, Goals, Satisfaction, and Transition to Home. *J Neurol Phys Ther*. 2019;43(3):160-167. doi:10.1097/NPT.0000000000000281 | Wrong Population (age (<18), stroke more than 5 years ago, health care professionals) |
| Lawrence M, Kerr S, Watson H, Paton G, Ellis G. An exploration of lifestyle beliefs and lifestyle behaviour following stroke: findings from a focus group study of patients and family members. *BMC Fam Pract*. 2010;11:97. doi:10.1186/1471-2296-11-97 | Focus on general needs, information satisfaction or education |
| Lefebvre H, Levert MJ, Pelchat D, Lepage JG. Nature, sources, and impact of information on the adjustment of family caregivers: a pilot project. *Can J Nurs Res = Rev Can Rech en Sci Infirm*. 2008;40(1):143-160. | Study size (n<8 quali; n<30 quanti) |
| Li X, Xia X, Wang P, Zhang S, Liu M, Wang L. Needs and rights awareness of stroke survivors and caregivers: a cross-sectional, single-centre questionnaire survey. *BMJ Open*. 2017;7(10):e013210-e013210. doi:10.1136/bmjopen-2016-013210 | Wrong Setting (developing countries) |
| Liddle J, Turpin M, McKenna K, Kubus T, Lambley S, McCaffrey K. The experiences and needs of people who cease driving after stroke. *Brain Impair*. 2009;10(3):271-281. doi:10.1375/brim.10.3.271 | Wrong Population (age (<18), stroke more than 5 years ago, health care professionals) |
| Lin B, Zhang Z, Guo Y, et al. Perceptions of recurrence risk and behavioural changes among first-ever and recurrent stroke survivors: A qualitative analysis. *Heal Expect an Int J public Particip Heal care Heal policy*. 2021;24(6):1962-1970. doi:10.1111/hex.13335 | Wrong Setting (developing countries) |
| LL P, Steiner V, JR S, Wicks B, Wright C, Thompson T. Questions caregivers asked in caring for persons with stroke. *Online J Nurs Informatics*. 2010;14(2):1-20. | Focus on general needs, information satisfaction or education |
| Lobo EH, Frølich A, Abdelrazek M, et al. Information, involvement, self-care and support-The needs of caregivers of people with stroke: A grounded theory approach. *PLoS One*. 2023;18(1):e0281198-e0281198. doi:10.1371/journal.pone.0281198 | Wrong Study design (Conference abstracts, reviews) |
| Low JTS, Kersten P, Ashburn A, George S, McLellan DL. A study to evaluate the met and unmet needs of members belonging to Young Stroke groups affiliated with the Stroke Association. *Disabil Rehabil*. 2003;25(18):1052-1056. doi:10.1080/0963828031000069753 | Wrong Population (age (<18), stroke more than 5 years ago, health care professionals) |
| Lui MH, MacKenzie AE. Chinese elderly patients’ perceptions of their rehabilitation needs following a stroke. *J Adv Nurs*. 1999;30(2):391-400. doi:10.1046/j.1365-2648.1999.01087.x | Wrong Setting (developing countries) |
| Mackenzie A, Perry L, Lockhart E, Cottee M, Cloud G, Mann H. Family carers of stroke survivors: needs, knowledge, satisfaction and competence in caring. *Disabil Rehabil*. 2007;29(2):111-121. doi:10.1080/09638280600731599 | Focus on general needs, information satisfaction or education |
| Malewezi E, O’Brien MR, Knighting K, Thomas J, Jack B. A different way of life: a qualitative study on the experiences of family caregivers of stroke survivors living at home. *Br J Community Nurs*. 2022;27(11):558-566. doi:10.12968/bjcn.2022.27.11.558 | Wrong Population (age (<18), stroke more than 5 years ago, health care professionals) |
| McLean J, Roper-Hall A, Mayer P, Main A. Service needs of stroke survivors and their informal carers: a pilot study. *J Adv Nurs*. 1991;16(5):559-564. doi:10.1111/j.1365-2648.1991.tb01691.x | Focus on general needs, information satisfaction or education |
| Moroni L, Sguazzin C, Filipponi L, et al. Caregiver Need Assessment: uno strumento di analisi dei bisogni del caregiver [Caregiver Need Assessment: a questionnaire for caregiver demand]. *G Ital Med Lav Ergon*. 2008;30(3 Suppl B):B84-B90. | Language (not in English or German) |
| Murtagh MJ, Burges Watson DL, Jenkings KN, et al. Situationally-sensitive knowledge translation and relational decision making in hyperacute stroke: a qualitative study. *PLoS One*. 2012;7(6):e37066-e37066. doi:10.1371/journal.pone.0037066 | Focus on general needs, information satisfaction or education |
| O’Mahony PG, Rodgers H, Thomson RG, Dobson R, James OF. Satisfaction with information and advice received by stroke patients. *Clin Rehabil*. 1997;11(1):68-72. doi:10.1177/026921559701100110 | Focus on general needs, information satisfaction or education |
| Nydevik I, Eller B. Stroke patients in long-term care--the relatives' conception of functional capacity and appropriate care. *Scand J Caring Sci*. 1994;8(3):155-161. doi:10.1111/j.1471-6712.1994.tb00014.x | Focus on general needs, information satisfaction or education |
| Pettersen S, Bøe MG, Haraldstad K. «Frisk, men likevel ikke som før...» Slagrammedes erfaringer etter hjerneslag og trombolytisk behandling. *Nord Nurs Res / Nord Sygeplejeforskning*. 2017;7(2):90-103. doi:10.18261/ISSN.1892-2686-2017-02-02 | Language (not in English or German) |
| RJ H, Brashler R. Assessment of the needs of the young stroke survivor. *Top Stroke Rehabil*. 1994;1(1):15-24. doi:10.1080/10749357.1994.11754003 | Wrong Population (age (<18), stroke more than 5 years ago, health care professionals) |
| Rochette A, Thomas A, Salbach NM, et al. Expected Health Benefits as the Ultimate Outcome of Information Available on Stroke Engine, a Knowledge Translation Stroke Rehabilitation Website: Web-Based Survey. *JMIR Rehabil Assist Technol*. 2023;10:e44715-e44715. doi:10.2196/44715 | Wrong Population (age (<18), stroke more than 5 years ago, health care professionals) |
| Röding J, Glader EL, Malm J, Eriksson M, Lindström B. Perceived impaired physical and cognitive functions after stroke in men and women between 18 and 55 years of age--a national survey. *Disabil Rehabil*. 2009;31(13):1092-1099. doi:10.1080/09638280802510965 | Focus on general needs, information satisfaction or education |
| Röding J, Lindström B, Malm J, Ohman A. Frustrated and invisible--younger stroke patients’ experiences of the rehabilitation process. *Disabil Rehabil*. 2003;25(15):867-874. doi:10.1080/0963828031000122276 | Study size (n<8 quali; n<30 quanti) |
| Rothwell K, Boaden R, Bamford D, Tyrrell PJ. Feasibility of assessing the needs of stroke patients after six months using the GM-SAT. *Clin Rehabil*. 2013;27(3):264-271. doi:10.1177/0269215512457403 | Focus on general needs, information satisfaction or education |
| Roy, D., Gasquoine, S., Caldwell, S., & Nash, D. (2015). Health professional and family perceptions of post-stroke information. Nursing Praxis in New Zealand, 31(2), pp.7-24 | Focus on general needs, information satisfaction or education |
| Salonen T. Report of a questionnaire survey of poststroke patients with aphasia and their families. *Top Stroke Rehabil*. 1995;2(3):72-75. doi:10.1080/10749357.1995.11754082 | Focus on general needs, information satisfaction or education |
| Shipley J, Luker J, Thijs V, Bernhardt J. How can stroke care be improved for younger service users? A qualitative study on the unmet needs of younger adults in inpatient and outpatient stroke care in Australia. *Disabil Rehabil*. 2020;42(12):1697-1704. doi:10.1080/09638288.2018.1534278 | Wrong Population (age (<18), stroke more than 5 years ago, health care professionals) |
| Sidek NN, Kamalakannan S, Tengku Ismail TA, et al. Experiences and needs of the caregivers of stroke survivors in Malaysia-A phenomenological exploration. *Front Neurol*. 2022;13:996620. Published 2022 Sep 23. doi:10.3389/fneur.2022.996620 | Wrong Setting (developing countries) |
| Simon C, Kendrick T. Community provision for informal live-in carers of stroke patients. *Br J Community Nurs*. 2002;7(6):292-298. doi:10.12968/bjcn.2002.7.6.10473 | Wrong Population (age (<18), stroke more than 5 years ago, health care professionals) |
| Smith M, Rousseau N, Lecouturier J, Gregson B, Bond J, Rodgers H. Are older people satisfied with discharge information?. *Nurs Times*. 1997;93(43):52-53. | Wrong Study design (Conference abstracts, reviews) |
| Slot KB, Berge E. Thrombolytic treatment for stroke: patient preferences for treatment, information, and involvement. *J stroke Cerebrovasc Dis Off J Natl Stroke Assoc*. 2009;18(1):17-22. doi:10.1016/j.jstrokecerebrovasdis.2008.06.009 | Focus on general needs, information satisfaction or education |
| Stein J, Hillinger M, Clancy C, Bishop L. Sexuality after stroke: patient counseling preferences. *Disabil Rehabil*. 2013;35(21):1842-1847. doi:10.3109/09638288.2012.754953 | Wrong Population (age (<18), stroke more than 5 years ago, health care professionals) |
| Sumathipala K, Radcliffe E, Sadler E, Wolfe CDA, McKevitt C. Identifying the long-term needs of stroke survivors using the International Classification of Functioning, Disability and Health. *Chronic Illn*. 2012;8(1):31-44. doi:10.1177/1742395311423848 | Wrong Population (age (<18), stroke more than 5 years ago, health care professionals) |
| Sutton KM, Kitzman PH, Hunter EG, et al. Engaging Individuals with Neurological Conditions and Caregivers in Rural Communities in a Health Research Team. *Prog Community Health Partnersh*. 2019;13(2):129-139. doi:10.1353/cpr.2019.0027 | Not on stroke (other diseases) |
| Tamrat EG, Gufue ZH, Getachew S, Yifru YM, Gizaw M. Factors associated with the longer-term unmet supportive care needs of stroke survivors in Ethiopia: a multicentre cross-sectional study. *BMJ Open*. 2022;12(1):e053579. Published 2022 Jan 31. doi:10.1136/bmjopen-2021-053579 | Wrong Setting (developing countries) |
| Tsai PC, Yip PK, Tai JJ, Lou MF. Needs of family caregivers of stroke patients: a longitudinal study of caregivers’ perspectives. *Patient Prefer Adherence*. 2015;9:449-457. doi:10.2147/PPA.S77713 | Wrong Setting (developing countries) |
| Turner GM, McMullan C, Atkins L, Foy R, Mant J, Calvert M. TIA and minor stroke: a qualitative study of long-term impact and experiences of follow-up care. *BMC Fam Pract*. 2019;20(1):176. doi:10.1186/s12875-019-1057-x | Focus on general needs, information satisfaction or education |
| van Til JA, Drossaert CHC, Renzenbrink GJ, et al. Feasibility of web-based decision aids in neurological patients. *J Telemed Telecare*. 2010;16(1):48-52. doi:10.1258/jtt.2009.001012 | Focus on general needs, information satisfaction or education |
| Visvanathan A, Mead GE, Dennis M, Whiteley WN, Doubal FN, Lawton J. The considerations, experiences and support needs of family members making treatment decisions for patients admitted with major stroke: a qualitative study. *BMC Med Inform Decis Mak*. 2020;20(1):98. doi:10.1186/s12911-020-01137-7 | Focus on general needs, information satisfaction or education |
| von Renteln-Kruse W, Nogaschewski K, Meier-Baumgartner HP. [Knowledge concerning illness, expectations and perceptions of treatment of elderly stroke patients and family caregivers--a prospective study during inpatient treatment]. *Z Gerontol Geriatr*. 2002;35(3):241-249. doi:10.1007/s00391-002-0051-3 | Focus on general needs, information satisfaction or education |
| Wachters-Kaufmann C, Schuling J, The H, Meyboom-de Jong B. Actual and desired information provision after a stroke. *Patient Educ Couns*. 2005;56(2):211-217. doi:10.1016/j.pec.2004.02.012 | Focus on general needs, information satisfaction or education |
| Wellwood I, Dennis M, Warlow C. Patients’ and carers’ satisfaction with acute stroke management. *Age Ageing*. 1995;24(6):519-524. doi:10.1093/ageing/24.6.519 | Focus on general needs, information satisfaction or education |
| Weppner DM, Brownscheidle CM. The evaluation of the health care needs of women with disabilities. *Prim Care Update Ob Gyns*. 1998;5(4):210. doi:10.1016/s1068-607x(98)00152-8 | Wrong Study design (Conference abstracts, reviews) |

**sTable 3 Detailed overview of the frequency of the domains of information requirements and their subdomains with corresponding references**

| Domain | Subdomains | Frequency (%) |
| --- | --- | --- |
| Treatment (n=28)  ^1–28^ | Acute treatment ^1,5,9,12–16,19,20,25^ | 11 (39.3%) |
|  | Drug treatment ^3,5,7–9,13,19,22,24,27^ | 10 (35.7%) |
|  | Further treatment  ^1,2,4–6,9–11,17,18,21,26,27^ | 13 (46.4% |
|  | Alternative treatment^23^ | 1 (3.6%) |
|  | Risk and benefits ^28^ | 1 (3.6%) |
| Etiology of stroke (n=23)  ^1–3,5–9,11,15,17,18,20,21,24–32^ | Characteristics of stroke ^3,6–9,11,15,17,18,20,21,24,26–30^ | 17 (73.9%) |
|  | Symptoms of stroke ^25,32^ | 2 (8.7%) |
|  | Risk factors^2,6,9,17,18^ | 5 (21.7%) |
|  | Warning signs^32^ | 1 (4.3%) |
|  | Diagnosis of stroke^2,5,18^ | 3 (13.0%) |
| Personalized information (n=19)  ^2,4,6,9,10,12–14,19,21,22,24,26–29,33,34^ | Specific information about patients’ stroke ^6,9,10,12–14,19–22,24,27,29,33,34^ | 15 (78.9%) |
|  | Comparison with other ^4,28^ | 2 (10.5%) |
| Community/social support (n=18)  ^4,5,7–11,14,17,20–22,24,26,29–31,33^ | Legal information following stroke  ^4,9,10,14,17,19,21,30^ | 8 (44.4%) |
|  | Statutory support  ^5,7–9,17,21,22,24,26,30^ | 10 (55.6%) |
|  | Informal support  ^9,17,26^ | 3 (16.7%) |
| Prognosis (n=18)  ^1,4–6,9–11,14,17,19,21,23,24,29,30,33,35,36^ | Timeline of recovery  ^4–6,21,23,33^ | 6 (33.3%) |
|  | Degree of recovery  ^4–6,9,21,30,36^ | 7 (38.8%) |
|  | Specific facts concerning the patient's progress  ^14^ | 1 (5.5%) |
| Effects of stroke (n=15)  ^1,4,5,7–9,11,14,15,17,19,21,28–30^ | Physical effects of stroke  ^1,4,7–9,17^ | 6 (40.0%) |
|  | Cognitive effect of stroke  ^7,9,17,30^ | 4 (26.7%) |
|  | Emotional/psychological impact of stroke  ^4,5,9,11,29,30^ | 6 (40.0%) |
|  | Behavioral effects of stroke  ^9,30^ | 2 (13.3%) |
|  | Effects on family and/or marriage  ^7–9,17^ | 4 (26.7%) |
| Care role (n=12)  ^7–9,12,14,19,21,22,24,25,29,32^ | Care for patients at home ^7,8,22,25,32^ | 5 (41.7%) |
|  | How to help with patients' physical care^7,8,12,14^ | 4 (33.3%) |
| Rehabilitation (n=11)  ^6,9,10,13,17–19,21–24^ | Expectations  ^6,9,17^ | 3 (27.3%) |
|  | Information about the rehabilitation units  ^23^ | 1 (9.1%) |
|  | Rehabilitation at home  ^9,10,17,19,21^ | 5 (45.5%) |
| Emotional support (n=10)  ^7–9,11,17,18,21,28,29,35^ | Emotional recollections of the event  ^28,35^ | 2 (20.0%) |
|  | Coping strategies  ^7–9,11,18,21,29^ | 7 (70.0%) |
| Transfer/Discharge (n=5)  ^1,4,5,17,30^ | Transfer to other units  ^1^ | 1 (20.0%) |
|  | Discharge home  ^1,4^ | 2 (40.0%) |
| Recommended lifestyle changes (n=5)  ^1,9,26,31,32^ | Healthy living  ^1,9,32^ | 3 (60.0%) |
| Information source (n=3)  ^4,9,30^ | Places that can help me and where to find more information  ^4,9,30^ | 3 (100%) |
| Hospital process (n=3)  ^4,14,15^ | Ward rules, responsibilities, procedures  ^4,14,15^ | 3 (100%) |

**sTabele 4 Information needs stratified by time period after the stroke (n=20)**

|  | **Acute phase (n=3)** | **Discharge phase (n=4)** | **Rehabilitation phase (n=6)** | **Post-stroke phase (n=7)** | **Total**  **(n=20)** |
| --- | --- | --- | --- | --- | --- |
| **Treatment** | 3 | 4 | 4 | 4 | 15 |
| **Etiology of stroke** | 2 | 1 | 5 | 4 | 12 |
| **Personalized information** | 2 | 4 | 0 | 3 | 9 |
| **Community/social support** | 1 | 2 | 3 | 5 | 11 |
| **Prognosis** | 1 | 0 | 2 | 3 | 6 |
| **Effects of stroke** | 3 | 0 | 2 | 3 | 8 |
| **Care role** | 1 | 2 | 1 | 2 | 6 |
| **Rehabilitation** | 0 | 2 | 2 | 1 | 5 |
| **Emotional support** | 1 | 0 | 2 | 2 | 5 |
| **Transfer/Discharge** | 0 | 0 | 1 | 1 | 2 |
| **Lifestyle** | 0 | 0 | 2 | 1 | 3 |
| **Information source** | 0 | 0 | 0 | 1 | 1 |
| **Hospital process** | 1 | 0 | 0 | 0 | 1 |

References

1. Abrahamson V, Wilson PM. How unmet are unmet needs post-stroke? A policy analysis of the six-month review. *BMC Health Serv Res* 2019; 19: 480.

2. Allison R, Evans PH, Kilbride C, et al. Secondary prevention of stroke: using the experiences of patients and carers to inform the development of an educational resource. *Fam Pract* 2008; 25: 355–361.

3. Donnellan C, Martins A, Conlon A, et al. Mapping patients’ experiences after stroke onto a patient-focused intervention framework. *Disabil Rehabil* 2013; 35: 483–491.

4. Finch E, Minchell E, Cameron A, et al. What do stroke survivors want in stroke education and information provision in Australia? *Health Soc Care Community* 2022; 30: e4864–e4872.

5. Garrett D, Cowdell F. Information needs of patients and carers following stroke. *Nurs Older People* 2005; 17: 14–16.

6. Hanger H, Walker G, Paterson L, et al. What do patients and their carers want to know about stroke? A two-year follow-up study. *Clin Rehabil* 1998; 12: 45–52.

7. Hinojosa MS, Rittman MR. Stroke caregiver information needs: comparison of Mainland and Puerto Rican caregivers. *J Rehabil Res Dev* 2007; 44: 649–658.

8. Hinojosa MS, Rittman M. Association between health education needs and stroke caregiver injury. *J Aging Health* 2009; 21: 1040–1058.

9. Hoffmann TC, McKenna K, Worrall L, et al. Evaluating current practice in the provision of written information to stroke patients and their carers. *Int J Ther Rehabil* 2004; 11: 303–310.

10. Lamontagne M-E, Richards C, Azzaria L, et al. Perspective of patients and caregivers about stroke rehabilitation: the Quebec experience. *Top Stroke Rehabil* 2019; 26: 39–48.

11. Perry L, Middleton S. An investigation of family carers’ needs following stroke survivors’ discharge from acute hospital care in Australia. *Disabil Rehabil* 2011; 33: 1890–1900.

12. Rosenthal SG, Pituch MJ, Greninger LO, et al. Perceived needs of wives of stroke patients. *Rehabil Nurs Off J Assoc Rehabil Nurses* 1993; 18: 148-153,167.

13. Almborg A-H, Ulander K, Thulin A, et al. Patients’ perceptions of their participation in discharge planning after acute stroke. *J Clin Nurs* 2009; 18: 199–209.

14. van der Smagt-Duijnstee ME, Hamers JP, Abu-Saad HH, et al. Relatives of hospitalized stroke patients: their needs for information, counselling and accessibility. *J Adv Nurs* 2001; 33: 307–315.

15. van der Smagt-Duijnstee ME, Hamers JP, Abu-Saad HH. Relatives of stroke patients--their experiences and needs in hospital. *Scand J Caring Sci* 2000; 14: 44–51.

16. Souter C, Kinnear A, Kinnear M, et al. Optimisation of secondary prevention of stroke: a qualitative study of stroke patients’ beliefs, concerns and difficulties with their medicines. *Int J Pharm Pract* 2014; 22: 424–432.

17. Tooth L, Hoffmann T. Patient Perceptions of the Quality of Information Provided in a Hospital Stroke Rehabilitation Unit. *Br J Occup Ther* 2004; 67: 111–117.

18. van Veenendaal H, Grinspun DR, Adriaanse HP. Educational needs of stroke survivors and their family members, as perceived by themselves and by health professionals. *Patient Educ Couns* 1996; 28: 265–276.

19. Wallengren C, Segesten K, Friberg F. Relatives’ information needs and the characteristics of their search for information--in the words of relatives of stroke survivors. *J Clin Nurs* 2010; 19: 2888–2896.

20. Wellwood I, Dennis MS, Warlow CP. Perceptions and knowledge of stroke among surviving patients with stroke and their carers. *Age Ageing* 1994; 23: 293–298.

21. Wiles R, Pain H, Buckland S, et al. Providing appropriate information to patients and carers following a stroke. *J Adv Nurs* 1998; 28: 794–801.

22. Almborg A-H, Ulander K, Thulin A, et al. Discharge planning of stroke patients: the relatives’ perceptions of participation. *J Clin Nurs* 2009; 18: 857–865.

23. Bamm EL, Rosenbaum P, Wilkins S, et al. Exploring Client-Centered Care Experiences in In-Patient Rehabilitation Settings. *Glob Qual Nurs Res* 2015; 2: 2333393615582036–2333393615582036.

24. Berg A, Tapiola T, Hujala M. Spouses’ need for information and satisfaction with the patient’s care and rehabilitation after stroke. Importance of depression and prescheduled follow-up. *Patient Educ Couns* 2023; 107: 107589.

25. Camicia M, Lutz BJ, Markoff N, et al. Determining the Needs of Family Caregivers of Stroke Patients During Inpatient Rehabilitation Using Interview, Art, and Survey. *Rehabil Nurs* 2019; 44: 328–337.

26. Cobley CS, Fisher RJ, Chouliara N, et al. A qualitative study exploring patients’ and carers’ experiences of Early Supported Discharge services after stroke. *Clin Rehabil* 2013; 27: 750–757.

27. Croot EJ, Ryan TW, Read J, et al. Transient ischaemic attack: a qualitative study of the long term consequences for patients. *BMC Fam Pract* 2014; 15: 174.

28. Decker C, Chhatriwalla E, Gialde E, et al. Patient-Centered Decision Support in Acute Ischemic Stroke: Qualitative Study of Patients’ and Providers’ Perspectives. *Circ Cardiovasc Qual Outcomes* 2015; 8: S109-16.

29. O’Connell B, Baker L, Prosser A. The educational needs of caregivers of stroke survivors in acute and community settings. *J Neurosci Nurs J Am Assoc Neurosci Nurses* 2003; 35: 21–28.

30. Kerr J, Hilari K, Litosseliti L. Information needs after stroke: What to include and how to structure it on a website. A qualitative study using focus groups and card sorting. *Aphasiology* 2010; 24: 1170–1196.

31. Eames S, Hoffmann T, Worrall L, et al. Delivery styles and formats for different stroke information topics: patient and carer preferences. *Patient Educ Couns* 2011; 84: e18-23.

32. Bakas T, Austin JK, Okonkwo KF, et al. Needs, concerns, strategies, and advice of stroke caregivers the first 6 months after discharge. *J Neurosci Nurs J Am Assoc Neurosci Nurses* 2002; 34: 242–251.

33. Kristensson L, Björkdahl A. Experience of Information Provision at the Stroke Unit From the Perspective of Relatives to Stroke Survivors. *Rehabil Process outcome* 2020; 9: 1179572720947086–1179572720947086.

34. Dalli LL, Andrew NE, Kim J, et al. Understanding of medications and associations with adherence, unmet needs, and perceived control of risk factors at two years post-stroke. *Res Social Adm Pharm* 2022; 18: 3542–3549.

35. Visvanathan A, Mead G, Dennis M, et al. Maintaining hope after a disabling stroke: A longitudinal qualitative study of patients’ experiences, views, information needs and approaches towards making treatment decisions. *PLoS One* 2019; 14: e0222500–e0222500.

36. von Vogelsang A-C, Nymark C, Pettersson S, et al. ‘My head feels like it has gone through a mixer’ - a qualitative interview study on recovery 1 year after aneurysmal subarachnoid hemorrhage. *Disabil Rehabil* 2023; 45: 1323–1331.
